# Femoral hernia caused by endometriosis in femoral lymph nodes: a rare case report

**DOI:** 10.1093/jscr/rjaf773

**Published:** 2025-09-28

**Authors:** Ayşe Sena Çalış

**Affiliations:** Department of General Surgery, Ministry of Health Sungurlu State Hospital, Yenihayat Street, Sungurlu District, Çorum 19300, Türkiye

**Keywords:** femoral hernia, endometriosis, hernia repair, lymph nodes

## Abstract

Endometriosis is a chronic, estrogen-dependent disease commonly affecting pelvic organs. Lymph node involvement is rare and even more so when leading to herniation. We report a rare case of a 41-year-old woman with no prior history of endometriosis, who presented with right groin pain and a palpable mass. Imaging suggested a femoral hernia. During open surgical repair, enlarged lymph nodes were discovered in the femoral canal causing femoral hernia. Histopathological analysis revealed endometriotic tissue within the lymph nodes. This case highlights a rare extrapelvic manifestation of endometriosis causing a femoral hernia. Endometriosis should be considered in the differential diagnosis of groin masses, especially in women of reproductive age with cyclical symptoms.

## Introduction

Hernias of the groin, including inguinal and femoral types, are common clinical conditions. Femoral hernias are more prevalent in women and often misdiagnosed as inguinal hernias due to overlapping symptoms. They can present with groin discomfort, a palpable mass, and, in rare cases, bowel obstruction or incarceration [1–3].

Endometriosis is a chronic, estrogen-dependent disorder affecting up to 10% of women of reproductive age. It typically involves the pelvic peritoneum, ovaries, and rectovaginal septum. Extrapelvic endometriosis, including lymphatic spread to inguinal or femoral lymph nodes, is extremely rare [4, 5]. This case report describes a unique presentation of femoral hernia caused by endometriotic involvement of femoral lymph nodes.

## Case report

A 41-year-old multiparous woman with no history of endometriosis presented to a general surgery clinic with complaints of right-sided pelvic pain and a palpable groin mass. The pain worsened cyclically during menstruation. Physical examination revealed a suspected femoral hernia, and ultrasonography confirmed the presence of a right femoral hernia without incarceration.

Given the cyclical nature of pain, the patient was also evaluated by a gynaecologist. A transvaginal ultrasound performed 15 days prior to surgery showed no evidence of deep pelvic endometriosis or ovarian abnormalities. The patient was using an intrauterine device for contraception.

She was admitted for elective open femoral hernia repair under spinal anesthesia. Intraoperatively, enlarged and palpable lymph nodes were found within the femoral canal. The patient was asked to perform a Valsalva maneuver, which revealed herniation of lymph nodes through the femoral canal. No bowel or other visceral structures were involved. Two lymph nodes were excised for histopathological examination, and the hernia was repaired using mesh hernioplasty.

**Figure 1:**
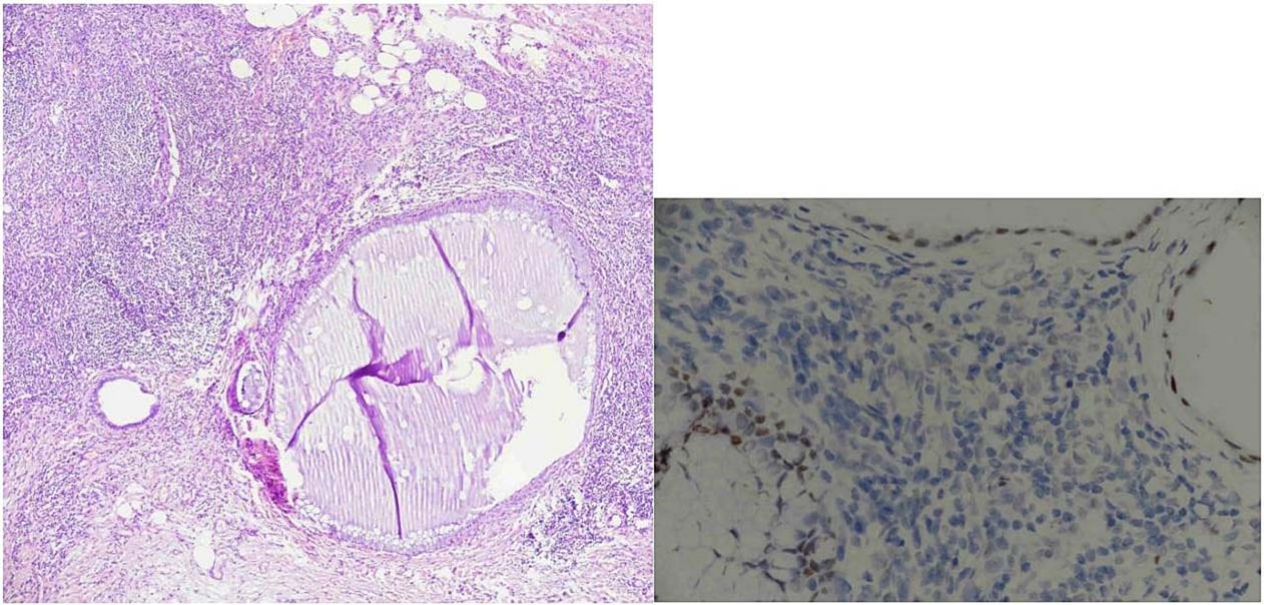
Lymph node endometriosis. Lymph node number 1, A: Hematoxylin/eosin stain × 40; B: Estrogen receptor immunostaining × 200.

**Figure 2:**
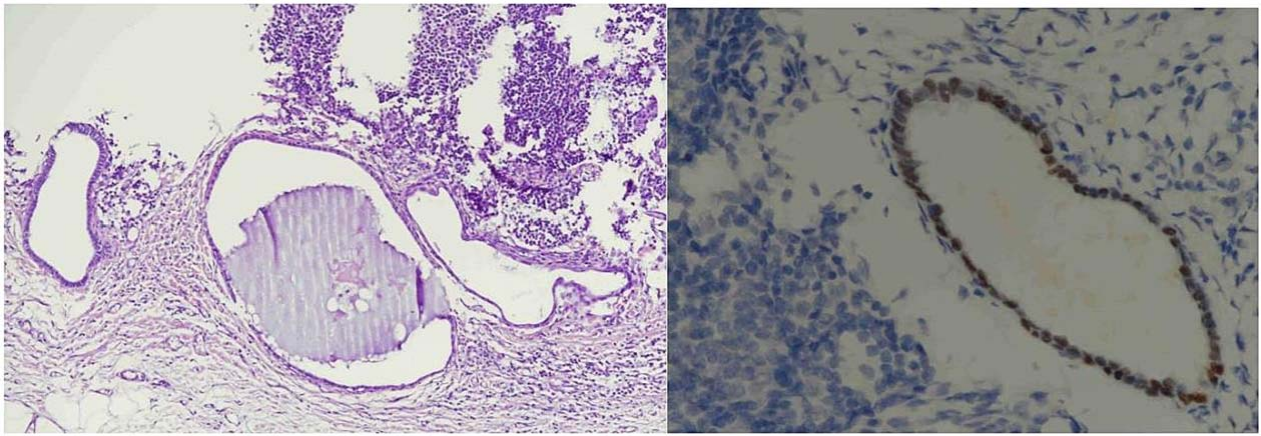
Lymph node endometriosis. Lymph node number 2, A: Hematoxylin/eosin stain × 100; B: Estrogen receptor immunostaining × 400.

Histopathological analysis showed two reactive lymph nodes measuring 2.0 cm and 3.5 cm. Immunohistochemistry demonstrated positivity for pancytokeratin, estrogen receptor (ER), and CD10, consistent with endometrial tissue. Histopathological images of the lymph nodes are presented in Figs 1 and 2. No signs of malignancy were identified.

The patient had an uneventful postoperative course and was discharged 1 day after surgery. At a mid-term follow-up, she reported complete resolution of pelvic pain with no signs of recurrence. A signed patient consent form for publication is on file at our institution.

## Discussion

Endometriosis involving femoral lymph nodes is extremely rare and can present with non-specific symptoms such as groin pain or a palpable mass. Most cases of inguinal or femoral endometriosis are secondary to pelvic disease or previous surgeries. However, in this case, the patient had no prior history or clinical evidence of pelvic endometriosis.

The mechanism of lymphatic spread in endometriosis remains controversial. Still, some theories suggest lymphatic and hematogenous dissemination in addition to retrograde menstruation [6].

This case is, to our knowledge, the first report of a femoral hernia caused by endometriotic lymph node enlargement in the absence of known pelvic endometriosis. Awareness of this rare entity can aid in the differential diagnosis of groin hernias, especially in women of reproductive age presenting with cyclical symptoms.

Endometriosis should be considered in the differential diagnosis of groin hernias, particularly in female patients presenting with cyclical pelvic pain. Lymphatic involvement by endometrial tissue, although rare, can lead to unusual presentations such as femoral herniation. Histopathological evaluation remains essential for diagnosis.
